# Screening-based low energy availability risk, disordered eating risk, and their co-occurrence among female volleyball players: a cross-sectional study

**DOI:** 10.1371/journal.pone.0360395

**Published:** 2026-10-08

**Authors:** Hülya André, Derya Çetin Sarışık, Arda Özçelik

**Affiliations:** 1 Department of Physical Education and Sports Education, Yozgat Bozok University, Yozgat, Türkiye; 2 Department of Physical Education and Sports, Bayburt University, Bayburt, Türkiye; 3 Department of Coaching Education, Yozgat Bozok University, Yozgat, Türkiye; Ondokuz Mayıs Üniversitesi: Ondokuz Mayis Universitesi, TÜRKIYE

## Abstract

Low energy availability (LEA) and disordered eating (DE) are important health concerns in female athletes, yet their occurrence and co-occurrence in team-sport contexts, particularly volleyball, remain underexplored. This cross-sectional online survey study examined the proportions of athletes classified as being at risk of LEA and DE overall and across competition levels, and assessed the co-occurrence between the two risk classifications in female volleyball players in Türkiye, using the Low Energy Availability in Females Questionnaire (LEAF-Q) and Eating Attitudes Test-26 (EAT-26). It also examined correlations between EAT-26 total score and LEAF-Q total and domain scores. A total of 213 athletes, recruited via non-probability convenience sampling, completed the LEAF-Q and EAT-26 to assess LEA risk and DE risk, respectively. In this sample, 33.3% of athletes were classified as being at risk of LEA, with no difference across competition levels (*p* = 0.825). A total of 17.4% were classified as being at risk of DE, with variation across competition levels and the highest proportion among Turkish Women’s Volleyball Second League athletes (*p* = 0.034). LEA risk and DE risk co-occurred in 10.3% of the sample, and DE risk was associated with higher odds of LEA risk (unadjusted OR = 3.80, 95% CI: 1.82–7.92). EAT-26 total score was positively correlated with LEAF-Q total and gastrointestinal function scores (*p* < 0.001) and injury history score (*p* = 0.004), but not menstrual function score (*p* = 0.133). These results suggest that LEA risk was commonly identified in this sample, DE risk was comparatively less common, and their co-occurrence was only partial, highlighting the value of screening for both risks rather than assuming one from the other.

## Introduction

Low energy availability (LEA) is an important concern in female athletes and occurs when the energy remaining after exercise-related energy expenditure is insufficient to support the physiological functions required to maintain health and performance [1]. Rather than having a single cause, LEA may arise from a mismatch between energy intake and exercise-related energy expenditure, shaped by training demands, nutritional barriers, body image concerns, psychosocial stressors, and the broader sport environment [2,3]. Depending on the duration and severity of this energy deficiency, its physiological consequences can range from transient and reversible to persistent and widespread. Short-term or less severe LEA that is associated with temporary and potentially reversible physiological changes is described as adaptable LEA. By contrast, problematic LEA refers to prolonged or severe exposure associated with persistent disturbances across reproductive, metabolic, immune, cardiovascular, gastrointestinal, psychological, and performance-related domains, often accompanied by signs and/or symptoms. Such exposure may ultimately contribute to the development of Relative Energy Deficiency in Sport (REDs), a broader syndrome characterized by impaired physiological and/or psychological functioning and detrimental health and performance outcomes [4].

Within this broader context, disordered eating (DE) may contribute to the development or persistence of LEA by reducing energy intake relative to training demands [2]. DE refers to a spectrum of maladaptive eating attitudes and behaviors, including restrictive dieting, fasting, binge eating, and inappropriate weight-control practices. Importantly, DE does not necessarily indicate a clinically diagnosed eating disorder; rather, it reflects subclinical or maladaptive eating-related attitudes and behaviors that may disrupt fueling practices and indicate clinically relevant eating-related risk when elevated [5]. In athletes, these behaviors may increase vulnerability to LEA, particularly when they occur alongside high training loads or inadequate nutritional support [2]. However, because LEA may also occur in the absence of DE when exercise-related energy expenditure is not adequately matched by dietary intake, the two should be considered distinct but potentially overlapping constructs rather than interchangeable terms [6].

Although research on LEA has predominantly focused on aesthetic, endurance, and weight-sensitive sports, studies using calculation-based [7,8] and screening-based [9,10] methods have also reported LEA and LEA risk, respectively, among female team-sport athletes. Volleyball-specific evidence suggests that inadequate dietary intake relative to training and competition demands, among other factors, may increase female volleyball players’ vulnerability to LEA. Supporting this concern, studies have reported energy and macronutrient intakes below estimated needs, as well as individual cases of calculated LEA [11,12]. Moreover, the prevalence of calculated LEA may vary across the competitive season [13]. Contextual factors may further increase this vulnerability, as gaps in sport nutrition knowledge and body image concerns associated with revealing uniforms may contribute to the broader context in which inadequate fueling can occur [2,11,14]. Beyond LEA, DE-related concerns have also been documented in volleyball, with dieting behaviors and elevated eating-related questionnaire scores reported among elite adolescent volleyball players [15], and screening-based DE risk identified among collegiate volleyball players [16]. Together, these findings underscore the relevance of examining both LEA risk and DE risk among female volleyball players.

Within this context, competition level represents a potentially relevant source of variation in LEA risk and DE risk among female volleyball players. Access to nutrition education and support resources may differ across competitive contexts [2]. Developmental stage may also be relevant, as younger athletes must meet the nutritional demands of both physical growth and athletic training [17]. However, available evidence is mixed and comes largely from non-volleyball samples. Among female athletes in aesthetic sports, screening-based LEA risk assessed using the Low Energy Availability in Females Questionnaire (LEAF-Q) was higher in elite than recreational athletes [18], whereas no significant difference was observed across competition levels among female combat-sport athletes [19]. For DE risk, one study using the Eating Attitudes Test-26 (EAT-26) reported more pathological eating attitudes at higher competition levels among female collegiate athletes [20], while another found an association with competition level among adolescents but not adults [21]. Given the differences in sport type, age group, and screening instrument across these studies, the findings do not support a clear directional expectation for female volleyball players.

In addition, volleyball-specific samples have generally been small, while evidence from broader athlete research often includes only limited numbers of volleyball players within mixed-sport samples [6,22,23]. Consequently, the extent to which screening-based LEA and DE risks co-occur among female volleyball players remains unclear, as does whether the prevalence of these risks differs across volleyball competition levels. Moreover, the LEAF-Q domains assess self-reported symptoms relating to gastrointestinal function, injury history, and menstrual function rather than directly measured energy availability [24], and their relationships with DE-related attitudes have not been well characterized in this population.

Therefore, this study aimed to determine the overall proportions of athletes classified as being at risk of LEA and DE, compare these proportions across competition levels, and assess the co-occurrence and statistical association between the two risk classifications among female volleyball players. The study also examined the associations between EAT-26 total score and LEAF-Q total and domain scores. We hypothesized that the proportions of athletes classified as being at risk of LEA and DE would each differ across competition levels, that the two risk classifications would co-occur and be positively associated, and that EAT-26 total score would correlate positively with LEAF-Q total score, though associations with individual domain scores might vary.

## Materials and methods

This study employed a cross-sectional design using a self-administered online survey. Recruitment and data collection took place between 17/05/2025 and 29/06/2025. A total of 213 female volleyball players participated. Participants competed in the Under-17 and Under-19 categories, combined into a single “Youth” group for analysis, the Turkish Women’s Volleyball Second League (TWV-2), and the Turkish Women’s Volleyball First League (TWV-1).

Participants were recruited using a non-probability convenience sampling approach. Volleyball coaches were contacted to facilitate access to eligible athletes, and interested athletes received the survey link from the research team. Eligibility criteria included being at least 15 years of age, having at least one year of licensed volleyball experience, regularly participating in team training sessions, and competing in official matches. Athletes with less than one year of licensed experience or no official competition history were excluded.

An a priori power analysis conducted using G*Power 3.1 indicated that a minimum sample size of 172 participants was required for chi-square tests comparing LEA risk and DE risk classifications across three competition levels, medium effect size w = 0.30, α = 0.05, power = 0.95, df = 2. For correlation analyses between the EAT-26 scores and the LEAF-Q total and domain-specific scores, a minimum of 139 participants was required, assuming a medium effect size of ρ = 0.30, α = 0.05, and power = 0.95. The final sample of 213 athletes exceeded both requirements.

To reduce potential response-related bias, the questionnaires were administered in a standardized, self-administered online format, and responses were collected anonymously. Nevertheless, convenience sampling and coach-facilitated recruitment may have introduced selection bias, while the concurrent collection of data using self-report questionnaires may have introduced common-method bias.

The study was approved by the Yozgat Bozok University Social and Human Sciences Ethics Committee, approval no: 25/31, dated 14 May 2025. All procedures were performed in accordance with the Declaration of Helsinki. Participation was voluntary, and electronic informed consent was obtained from all participants prior to accessing the survey. For athletes under 18 years of age, written parental or guardian consent and participant assent were also obtained.

### Measures

The online survey consisted of three sections: (1) a researcher-developed Personal Information Form, (2) the Low Energy Availability in Females Questionnaire (LEAF-Q), and (3) the Eating Attitudes Test-26 (EAT-26).

The Personal Information Form collected demographic and training-related data, including age, body mass, height, years of training experience, competition level, weekly training frequency, and session duration. Weekly training volume, expressed as hours per week, was calculated by multiplying weekly training frequency by session duration. Body mass index was computed using the standard formula, kg/m^2^.

LEA risk was assessed using the Turkish version of the LEAF-Q, which was originally developed by Melin et al. [24] and validated in Turkish by Çelenk Durgut et al. [25]. The LEAF-Q is a 25-item self-report screening instrument that identifies athletes at risk based on self-reported symptoms relating to three domains: gastrointestinal function, injury history, and menstrual function; it does not directly measure energy availability or confirm actual LEA status. Participants with a total score of ≥8 were classified as being at risk of LEA. Domain thresholds were ≥2 for gastrointestinal function, ≥ 2 for injury history, and ≥4 for menstrual function. The total score was used both for the categorical risk classification and as a continuous variable in the correlation analyses. The domain scores were examined separately in accordance with the study aim. The Turkish version demonstrated acceptable subscale internal consistency, Cronbach’s α = 0.617–0.744, and excellent test-retest reliability, intraclass correlation coefficient = 0.886. Although formal validation in athletes younger than 18 years remains limited, the LEAF-Q has previously been used in adolescent athletes, including female football players aged 13–18 years [10].

DE risk was assessed using the Turkish version of the EAT-26, which was originally developed by Garner et al. [26] and validated in Turkish by Ergüney Okumuş and Sertel Berk [27]. This 26-item self-report screening instrument does not establish a clinical diagnosis of an eating disorder. Participants with a total score of ≥20 were classified as being at risk of DE. It has also been used in adolescent athlete research, including as a comparator in a validation study involving high-school athletes [28]. Internal consistency was good in both the validation study, Cronbach’s α = 0.84, and the current sample, Cronbach’s α = 0.85. In the present analyses, its total score was used both as a continuous variable and to derive the categorical DE risk classification.

### Data analysis

Statistical analyses were performed using jamovi version 2.7.24 and R version 4.6.1 (R Core Team, 2026). Continuous variables are presented as mean ± standard deviation, and categorical variables as frequencies and percentages. The proportions of athletes classified as being at risk of LEA and DE were reported with 95% Wilson score confidence intervals. Associations between risk classifications and competition level were examined using Pearson’s chi-square test with Cramér’s V as the effect size. Significant omnibus tests were followed by exploratory pairwise Fisher’s exact tests with Holm adjustment for multiple comparisons. The co-occurrence of LEA and DE risk was examined using Pearson’s chi-square test and an unadjusted odds ratio with 95% confidence intervals. Normality was assessed using the Shapiro-Wilk test. Because EAT-26 and LEAF-Q scores were not normally distributed, Spearman’s rank-order correlations were used to examine associations between EAT-26 total score and LEAF-Q total and domain scores. A Bonferroni-adjusted alpha of 0.0125 was applied to the four correlation analyses; for all other analyses, statistical significance was set at two-sided *p* < 0.05. To assess whether the findings were sensitive to the inclusion of participants younger than 18 years, age-related sensitivity analyses were conducted. LEA and DE risk proportions, the association between DE risk and LEA risk, and the correlations between EAT-26 total score and LEAF-Q total and domain scores were compared between participants younger than 18 years and those aged 18 years or older. Differences in odds ratios and correlation coefficients between age groups were additionally examined using the Breslow-Day test and Fisher’s r-to-z transformation, respectively. Detailed results are presented in S1 Table.

## Results

The final analytic sample consisted of 213 female volleyball players, with no missing data for any variables included in the analyses. Participants were distributed across the Turkish Women’s Volleyball First League (TWV-1, n = 60), the Turkish Women’s Volleyball Second League (TWV-2, n = 110), and the Youth group, combined Under-17 and Under-19 categories, n = 43. Participant characteristics by competition level are presented in Table 1.

**Table 1:** Participant characteristics by competition level.

Variable	TWV-1 (n = 60)	TWV-2 (n = 110)	Youth (n = 43)	Total (N = 213)
**Age (years)**	22.18 ± 4.78	19.52 ± 4.41	16.40 ± 0.58	19.64 ± 4.51
**Height (cm)**	180.51 ± 7.70	175.55 ± 8.33	174.63 ± 8.71	176.76 ± 8.53
**Body mass (kg)**	66.69 ± 7.17	64.21 ± 8.95	66.45 ± 11.45	65.36 ± 9.11
**Body mass index (kg/m²)**	20.45 ± 1.63	20.80 ± 2.24	21.72 ± 2.94	20.89 ± 2.29
**Training experience (years)**	11.77 ± 4.24	8.73 ± 4.92	5.54 ± 2.48	8.94 ± 4.83
**Weekly training (h/week)**	16.20 ± 5.63	12.27 ± 5.40	10.41 ± 5.51	13.00 ± 5.86

Values are presented as mean ± standard deviation. TWV-1, Turkish Women’s Volleyball First League; TWV-2, Turkish Women’s Volleyball Second League; Youth, combined under-17 and under-19 categories.

Table 2 presents the proportions of athletes classified as being at risk of LEA and DE overall and by competition level, together with the results of the competition-level comparisons. LEA risk was not significantly associated with competition level, χ²(2) = 0.38, p = 0.825, Cramér’s V = 0.042. In contrast, DE risk was significantly associated with competition level, χ²(2) = 6.77, p = 0.034, Cramér’s V = 0.178. The highest DE risk proportion was observed among TWV-2 athletes, followed by Youth and TWV-1 athletes. Exploratory pairwise comparisons using Holm-adjusted Fisher’s exact tests showed that the DE risk proportion was significantly higher in TWV-2 than in TWV-1 athletes (adjusted p = 0.040), whereas the Youth group did not differ significantly from either group (both adjusted p = 0.537). In the age-related sensitivity analysis, LEA and DE risk proportions did not differ significantly between participants younger than 18 years and those aged 18 years or older (S1 Table).

**Table 2:** Proportions of athletes classified as being at risk of LEA and DE overall and by competition level.

Measure	TWV-1 (n = 60)	TWV-2 (n = 110)	Youth (n = 43)	Total (n = 213)	χ² (df)	*p*	Cramér’s V
**LEAF-Q**							
**At risk for LEA**	19 (31.7) [21.3-44.2]	36 (32.7) [24.7-41.9]	16 (37.2) [24.4-52.1]	71 (33.3) [27.3-39.9]	0.38 (2)	0.825	0.042
**Not at risk for LEA**	41 (68.3)	74 (67.3)	27 (62.8)	142 (66.7)			
**EAT-26**							
**At risk for DE**	5 (8.3)^a^ [3.6-18.1]	26 (23.6)^b^ [16.7-32.4]	6 (14.0)^a b^ [6.6-27.3]	37 (17.4) [12.9-23.0]	6.77 (2)	0.034	0.178
**Not at risk for DE**	55 (91.7)	84 (76.4)	37 (86.0)	176 (82.6)			

Values are n (% within competition level; 95% Wilson CI in brackets). LEA risk: LEAF-Q ≥ 8; DE risk: EAT-26 ≥ 20. Different superscript letters indicate a significant pairwise difference after Holm correction. DE, disordered eating; EAT-26, Eating Attitudes Test-26; LEA, low energy availability; LEAF-Q, Low Energy Availability in Females Questionnaire; TWV-1/TWV-2, Turkish Women’s Volleyball First/Second League; Youth, combined under-17/under-19 categories.

Table 3 presents the co-occurrence of LEA and DE risk and the association between them. A total of 22 athletes (10.3% of the sample) were classified as being at risk of both LEA and DE. Among athletes at risk of DE, 59.5% were also at risk of LEA, compared with 27.8% among those not at risk of DE. DE risk was associated with higher odds of LEA risk (unadjusted OR = 3.80, 95% CI [1.82–7.92], *p* < 0.001). The association was similar in direction and magnitude across age groups (S1 Table).

**Table 3:** Co-occurrence and association between LEA and DE risk.

LEAF-Q risk status	DE risk (n = 37)	No DE risk (n = 176)	Total (n = 213)	χ² (df)	Odds ratio [95% CI]	*p*
**At risk for LEA**	22 (59.5)	49 (27.8)	71 (33.3)	13.75 (1)	3.80 [1.82-7.92]	<0.001
**Not at risk for LEA**	15 (40.5)	127 (72.2)	142 (66.7)			
**Total**	37 (100.0)	176 (100.0)	213 (100.0)			

Values are n (% within DE risk status; % of total sample for the Total column). LEA risk: LEAF-Q ≥ 8; DE risk: EAT-26 ≥ 20. OR unadjusted. CI, confidence interval; DE, disordered eating; EAT-26, Eating Attitudes Test-26; LEA, low energy availability; LEAF-Q, Low Energy Availability in Females Questionnaire; OR, odds ratio.

Table 4 presents the associations between EAT-26 total score and LEAF-Q total and domain scores. EAT-26 total score showed a small-to-moderate positive correlation with the LEAF-Q total score (*r* = 0.279, *p* < 0.001) and small positive correlations with the gastrointestinal function domain score (*r* = 0.265, *p* < 0.001) and injury history domain score (*r* = 0.199, *p* = 0.004); all three associations remained significant at the Bonferroni-adjusted alpha level of 0.0125. The correlation with menstrual function domain score was not statistically significant (*r* = 0.103, *p* = 0.133). The pattern of correlations between EAT-26 total score and LEAF-Q total and domain scores was also broadly consistent across age groups (S1 Table).

**Table 4:** Associations between EAT-26 total score and LEAF-Q total and domain scores.

Variable	*r*	*p*
**LEAF-Q total score**	0.279	<0.001
**Gastrointestinal function domain score**	0.265	<0.001
**Injury history domain score**	0.199	0.004
**Menstrual function domain score**	0.103	0.133

Values are Spearman’s rank-order correlation coefficients. EAT-26, Eating Attitudes Test-26; LEAF-Q, Low Energy Availability in Females Questionnaire.

## Discussion

Overall, the findings provided partial support for the study hypotheses. Screening-based LEA risk and DE risk were identified in approximately one-third and one-sixth of the sample, respectively. LEA risk was not associated with competition level, whereas DE risk showed a small overall association with competition level, with exploratory post hoc comparisons indicating a higher proportion classified as being at risk among TWV-2 than TWV-1 athletes. The two risk classifications co-occurred and were positively associated, although their overlap was limited. EAT-26 total score was positively associated with LEAF-Q total score and the gastrointestinal function and injury history domain scores, whereas no significant association was observed with the menstrual function domain score.

Among the athletes in this sample, 33.3% were classified as being at risk of LEA, with no significant association with competition level. However, the study may have had limited power to detect small associations because the a priori power analysis was based on a medium effect size. Using the same LEAF-Q cut-off score, Logue et al. reported a screening-based LEA risk prevalence of 40% among 833 female athletes and recreationally active women [29], although the results were not reported separately by sport. Evidence specific to volleyball has more often been generated by calculating energy availability rather than through LEAF-Q screening. In Canadian female varsity volleyball players, 2 of 10 athletes had calculated energy availability below 30 kcal/kg fat-free mass/day [12]. Among female collegiate beach volleyball athletes, the prevalence below this threshold was 30.8% during preseason and ranged from 25.0% to 62.5% across in-season assessments [13]. Although these studies demonstrate that low calculated energy availability can occur in volleyball, their estimates should not be treated as direct prevalence benchmarks for the present LEAF-Q classification because the assessment methods, samples, and assessment periods differ. Methodological heterogeneity across the broader literature further limits comparisons between prevalence estimates [30]. Taken together, the available evidence indicates that energy availability warrants attention among female volleyball players while underscoring the need for further volleyball-specific research.

For DE risk, 17.4% of the athletes were classified as being at risk, with the highest proportion among TWV-2 athletes (23.6%), followed by Youth (14.0%) and TWV-1 (8.3%) athletes. DE risk was significantly associated with competition level, although the overall effect size was small. Post hoc pairwise comparisons indicated a higher proportion classified as being at risk among TWV-2 than TWV-1 athletes, whereas the Youth group did not differ significantly from either group. Because the specific pairwise contrasts were not prespecified and the analyses were not adjusted for age, body mass index, training volume, sport experience, or other potential confounders, the higher proportion observed among TWV-2 than TWV-1 athletes should be interpreted as exploratory rather than as an independent effect of competition level. Volleyball-specific evidence on DE risk remains scarce, limiting comparisons with existing literature. In a small sample of 14 female collegiate volleyball players, 50% met the EAT-26 risk threshold [16]. An earlier study using different measures also reported dieting behaviors and elevated eating-related questionnaire scores in 23 elite adolescent female volleyball players [15]. Although it did not provide a directly comparable EAT-26 prevalence estimate, the study adds to the limited evidence of eating-related concerns in female volleyball players. In broader female athlete samples, risk estimates obtained using the EAT-26 have ranged from 6.9% in highly trained adults and 14.6% in adolescents to 32.5% in Turkish university athletes [21,31]. The variation in risk estimates across these studies may reflect differences in sample characteristics, age, sport and competitive context, as well as the screening instrument and cut-off score used. Larger volleyball-specific studies using comparable screening methods and adjusted analyses are needed to determine whether DE risk differs meaningfully across competition levels.

The co-occurrence analysis extends these findings by showing a significant association between the two risk classifications in this sample. Athletes with DE risk had 3.80 times the odds of LEA risk compared with athletes without DE risk. However, only 22 athletes (10.3%) were classified as being at risk of both LEA and DE, whereas 49 were classified as being at risk of LEA but not DE. Thus, although the two risk classifications were associated, their overlap was limited. Because the odds ratio was unadjusted, this association should be interpreted cautiously and does not account for potential confounding. Previous studies suggest that eating-related risk or symptoms may be associated with LEAF-Q domain scores [6]. Low calculated energy availability has also been documented both with and without eating-disorder risk in a mixed collegiate sample that included indoor and beach volleyball players [23]. A similar pattern has been reported in recreational female runners [32], suggesting that the degree of co-occurrence may vary across samples and assessment methods.

Whereas the co-occurrence analysis examined the categorical LEA and DE risk classifications, the correlation analysis evaluated associations between EAT-26 total score and LEAF-Q total and domain scores. EAT-26 total score was positively associated with LEAF-Q total score (*r* = 0.279), representing a small-to-moderate relationship between DE-related attitudes and overall LEAF-Q symptom scores. This finding is consistent with previous studies reporting associations between disordered eating symptoms and LEAF-Q scores in female athlete samples, despite differences in eating-related screening tools [33,34].

At the domain level, EAT-26 total score showed small positive associations with the gastrointestinal function (*r* = 0.265) and injury history (*r* = 0.199) domain scores. The gastrointestinal finding is consistent with previous research showing that female athletes with disordered eating were more likely to report frequent gas and bloating [6]. Similarly, a small positive association between dietary restraint and gastrointestinal problem scores was reported among recreational female runners [32]. Evidence regarding injury-related outcomes is less consistent. No association between eating-disorder symptoms and injury was observed among recreational female runners [32], whereas studies involving adolescent female athletes have reported associations between eating-related risk and injury-related outcomes [35,36]. Because dietary intake, gastrointestinal physiology, and clinically verified injuries were not assessed, the present findings should be interpreted as associations between self-reported screening indicators rather than evidence of gastrointestinal pathology or injury causation.

In contrast, EAT-26 total score was not significantly associated with the menstrual function domain score. This finding suggests that the relationship between DE-related attitudes and LEAF-Q scores was not uniform across domains. Evidence regarding eating-related risk and menstrual function is mixed, though drawn from non-volleyball samples that are not directly comparable to the present cohort. Disordered eating and menstrual dysfunction have been shown to co-occur among high school female athletes [36], whereas a previous study using the LEAF-Q found no significant association between eating-disorder measures and menstrual function scores [32]. Menstrual function may also be influenced by factors beyond eating-related attitudes, including training load, hormonal contraceptive use, menstrual history, and individual physiological variability, which were not accounted for in these correlation analyses. Accordingly, the absence of a significant association in the present study should not be interpreted as evidence that menstrual function is unrelated to LEA risk. Rather, EAT-26 total score alone may not adequately reflect the factors associated with menstrual function in this sample.

Several limitations should be considered when interpreting these findings. The cross-sectional design precludes causal inference, and the concurrent administration of two self-report instruments may have introduced common-method bias. The LEAF-Q provides a screening-based risk classification rather than a direct measure of energy availability, while the EAT-26 does not establish a clinical diagnosis. Dietary intake, exercise energy expenditure, body composition, hormonal and bone-health markers, gastrointestinal physiology, and clinically verified injuries were not assessed, precluding direct examination of the mechanisms underlying the observed associations.

Convenience sampling, coach-facilitated recruitment, and the unknown response rate may have introduced selection bias and limited the representativeness of the observed risk proportions and competition-level comparisons; therefore, these findings should not be interpreted as population-level prevalence estimates. The analyses did not account for age, body mass index, training volume, sport experience, and other potential confounders, or within-team clustering; team membership was unavailable. Combining the Under-17 and Under-19 categories warrants caution when interpreting findings for the Youth group. In addition, participants younger than 18 years were represented across competition levels, and evidence regarding the psychometric performance of the Turkish versions of both instruments in this age group remains limited. Although the age-group sensitivity analyses showed broadly similar risk proportions and associations across age groups (S1 Table), this psychometric limitation remains. The absence of information on competitive-season phase and limited data on hormonal contraceptive use and menstrual status further constrain subgroup and domain-specific interpretations. Finally, the volleyball-specific focus of this study may limit generalizability of the findings to athletes from other sports or competitive systems.

The study also has several strengths. These include its sport-specific focus on female volleyball players, the inclusion of athletes from different competition levels, and the simultaneous assessment of LEA risk and DE risk within the same participants. This allowed the risk proportions, co-occurrence, and strength of association of both risk classifications to be examined within a single sample. In addition, the use of continuous LEAF-Q total and domain scores complemented the categorical risk classification and allowed overall and domain-specific relationships with the EAT-26 total score to be distinguished.

## Conclusion

The results of this study suggest that LEA risk and DE risk may be relevant health concerns among female volleyball players. In this sample, approximately one-third of the athletes were classified as being at risk of LEA**,** while approximately one-sixth were classified as being at risk of DE. LEA risk did not differ across competition levels, whereas DE risk was somewhat higher among TWV-2 athletes. Furthermore, the two risk classifications were positively associated, although their co-occurrence was limited. In addition, EAT-26 total score was positively associated with LEAF-Q total score and with the gastrointestinal function and injury history domain scores. In volleyball settings, the LEAF-Q and EAT-26 offer an easy-to-administer, brief, and low-cost approach to screening for both LEA and DE risk, within broader athlete-health monitoring. Screening may be accompanied by education for athletes and coaches on adequate fueling and the early warning signs of LEA and DE. A positive result could prompt individualized follow-up with appropriately qualified health professionals for further assessment where indicated. Because these instruments do not confirm LEA or DE, decisions concerning treatment or sport participation should be based on a more comprehensive assessment rather than screening results alone. Future research would benefit from larger and more representative samples, longitudinal assessments across the competitive season, the integration of screening instruments with objective and clinically verified measures, and analytical approaches that account for potential confounders and within-team clustering.

## Supporting information

S1 Dataset Anonymized analysis dataset and codebook. (XLSX)

S1 Table Age-group sensitivity analyses of LEA and DE risk classifications and their associations. (DOCX)
