# Supplementary material for: Screening-based low energy availability risk, disordered eating risk, and their co-occurrence among female volleyball players: a cross-sectional study
Source: PLoS One. 2026 Oct 8;21(10):e0360395. doi: 10.1371/journal.pone.0360395 (PMC13649128; doi:10.1371/journal.pone.0360395)
Supplement: Supplementary file 1 [file pone.0360395.s002.docx]

**S1 Table. Age-group sensitivity analyses of LEA and DE risk classifications and their associations.** Risk proportions, association between DE risk and LEA risk, and correlations between EAT-26 total score and LEAF-Q total and domain scores in participants younger than 18 years and those aged 18 years or older.

| **Measure** | **Age <18 years (n = 94)** | **Age ≥18 years (n = 119)** | **Test statistic** | ***p*** |
| --- | --- | --- | --- | --- |
| **A. Risk proportions, n (%) [95% Wilson CI]^a^** | | | | |
| At risk for LEA | 31 (33.0) [24.3-43.0] | 40 (33.6) [25.8-42.5] | χ²(1) = 0.01 | 0.922 |
| At risk for DE | 19 (20.2) [13.3-29.4] | 18 (15.1) [9.8-22.6] | χ²(1) = 0.95 | 0.331 |
| **B. Association between DE risk and LEA risk^b^** | | | | |
| At risk for LEA among athletes with DE risk, n/N (%) | 11/19 (57.9) | 11/18 (61.1) |  |  |
| At risk for LEA among athletes without DE risk, n/N (%) | 20/75 (26.7) | 29/101 (28.7) |  |  |
| Within-group association, χ²(1); *p* | 6.69; 0.010 | 7.19; 0.007 |  |  |
| Unadjusted OR [95% CI] | 3.78 [1.33-10.75] | 3.90 [1.38-11.05] | Breslow-Day χ²(1) < 0.01 | 0.967 |
| **C. Correlations between EAT-26 total score and LEAF-Q scores, *r* (*p*)^c^** | | | | |
| LEAF-Q total score | 0.262 (0.011) | 0.300 (<0.001) | *z* = −0.29 | 0.772 |
| Gastrointestinal function domain score | 0.297 (0.004) | 0.265 (0.004) | *z* = 0.25 | 0.803 |
| Injury history domain score | 0.156 (0.134) | 0.245 (0.007) | *z* = −0.67 | 0.504 |
| Menstrual function domain score | 0.092 (0.379) | 0.097 (0.292) | *z* = −0.04 | 0.968 |

Participants younger than 18 years were aged 16-17 years (Youth, n = 41; TWV-2, n = 45; TWV-1, n = 8). Participants aged 18 years or older were aged 18-36 years (Youth, n = 2; TWV-2, n = 65; TWV-1, n = 52). LEA risk was defined as a LEAF-Q total score ≥8 and DE risk as an EAT-26 total score ≥20.

^a^ Percentages are within age group. Between-group comparisons were performed using Pearson’s chi-square test.

^b^ Percentages are within DE risk status in each age group. Within-group associations were examined using Pearson’s chi-square test and unadjusted odds ratios with 95% confidence intervals. Homogeneity of odds ratios across age groups was examined using the Breslow-Day test.

^c^ Values are Spearman’s rank-order correlation coefficients. Within each age group, a Bonferroni-adjusted alpha of 0.0125 was applied to the four correlation analyses. Between-group differences in correlation coefficients were examined using Fisher’s *r*-to-*z* transformation.

These sensitivity analyses were exploratory and were not designed to establish equivalence between age groups. The modest subgroup sizes limited the power to detect small between-group differences, and the relatively small number of athletes classified as being at risk of DE in each age group (n = 19 and n = 18) further limited the precision of estimates involving DE risk. Therefore, nonsignificant between-group differences should be interpreted cautiously.

CI, confidence interval; DE, disordered eating; EAT-26, Eating Attitudes Test-26; LEA, low energy availability; LEAF-Q, Low Energy Availability in Females Questionnaire; OR, odds ratio; TWV-1, Turkish Women’s Volleyball First League; TWV-2, Turkish Women’s Volleyball Second League.
